# Factors predicting venous thromboembolism after spine surgery

**DOI:** 10.1097/MD.0000000000005776

**Published:** 2016-12-30

**Authors:** Tao Wang, Si-Dong Yang, Wen-Zheng Huang, Feng-Yu Liu, Hui Wang, Wen-Yuan Ding

**Affiliations:** a Department of Spinal Surgery, The Third Hospital of Hebei Medical University, Shijiazhuang; b Jizhong Energy Fengfeng Group General Hospital, Handan Shi, Hebei Province; c Hebei Provincial Key Laboratory of Orthopedic Biomechanics, Shijiazhuang, China.

**Keywords:** meta-analysis, predicted factor, spine surgery, venous thromboembolism

## Abstract

**Background:**

A meta-analysis was performed to explore predicted factors of venous thromboembolism (VTE) after surgery in the treatment for spine degeneration diseases.

**Summary of background data:**

Many scholars have focused on VTE after spine surgery, but as for the risk factors of VTE have not reached a consensus.

**Methods:**

An extensive search of literature, “spine or spinal,” “degeneration,” “after surgery or postoperation,” and “venous thromboembolism” as key words, was performed in PubMed/MEDLINE, Embase, the Cochrane library, CNKI, and WANFANG databases. The following variables were extracted: wearing elastic stocking, hypertension (HT), heart disease, diabetes, drinking, anticoagulant therapy, walking disability preoperation, smoking, sex, age, surgical duration, fusion versus nonfusion (lumbar fusion vs lumbar discectomy), surgical site (cervical vs lumbar), blood loss, and body mass index. Data analysis was conducted with RevMan 5.3 and STATA 12.0.

**Results:**

A total of 12 studies were identified, including 34,597 patients of whom 624 patients had VTE, and the incidence of VTE was 2% in all patients who underwent spine surgery. The incidence of VTE for Asian patients was 7.5%, compared with 1% VTE for Occidental patients; the difference was significant (*P* < 0.0001). The pooled analysis showed that there were significant differences regarding wearing elastic stocking (odds ratio [OR] = 11.71, 95% confidence interval [CI] [1.46, 94.00], *P* = 0.02), walking disability preoperation (OR = 4.80, 95% CI [2.53, 9.12], *P* < 0.00001), surgical site (lumbar surgery) (OR = 0.23, 95% CI [0.20, 0.27], *P* < 0.00001), HT (OR = 1.59, 95% CI [1.21, 2.10], *P* = 0.001), and diabetes (OR = 2.12, 95% CI [1.09, 4.10], *P* = 0.03). However, there were no significant differences in blood loss, heart disease, smoking, sex, surgical duration, body mass index, surgical duration, anticoagulant therapy, wearing elastic stocking, fusion versus nonfusion, drinking, and age (all *P* > 0.05).

**Conclusions:**

Based on our meta-analysis, Asian patients, patients with walking disability preoperation, patients wearing elastic stocking, patients having undergone lumbar surgery, patients with a history of HT, and patients experiencing diabetes have a higher incidence of VTE after spine surgery.

## 1 Introduction

Venous thromboembolism (VTE), including pulmonary embolism (PE) and deep vein thrombosis (DVT), is a common and potentially fatal disease.^[1]^ It may lead to severe morbidity with poor quality of life and even sudden death, bringing about serious burden to the patients and families. About half of all untreated DVT cases are complicated by PE, and, on the contrary, 50% to 80% of all untreated PE cases are related to DVT.^[2,3]^ Attention must be paid on VTE for surgeons, and increasing studies reported on risk factors of VTE.

Orthopedic surgery on the lower extremities, spinal cord injury, major trauma, and hip or leg fracture had a relatively high risk for VTE.^[4–6]^ Many studies reported on VTE after hip or knee arthroplasty.^[7–15]^ However, several studies focused on the risk factors of VTE in patients undergoing spine surgery^[3,8,15–20]^ and the incidence varied among these studies. Risk factors including long-time bed rest postoperatively and lack of lower limb activity are related to VTE after spine surgery. In surgical handling, venous intimal injury might occur.^[21–26]^ VTE is a common complication for patients after degenerative spine. A previous article reported that the rate of VTE was 15% for patients who underwent posterior spinal surgery without any prevention.^[27]^

Although VTE is commonly seen in patients undergoing spine surgery, to our knowledge, its incidence and related risk factors remain unclear. Hence, our study is aimed to explore the incidence of VTE and the risk factors associated with VTE for the patients who underwent spine surgery by a meta-analysis. Besides, we evaluate efficacy of using low-molecular-weight heparin after spine surgery and observe difference in incidence of VTE between Asians and the Occidentals.

## 2 Materials and methods

### 2.1 Ethics statement

It is not necessary to seek informed consent of patients, because our meta-analysis was based on published data and there is no potential harm to patients; this is approved by Ethics Committee of The Third Hospital of Hebei Medical University.

### 2.2 Search strategy

An extensive search of literature was performed, “deep venous thromboembolism,” “spine surgery,” and “spinal surgery” as key words, in PubMed, Embase, the Cochrane library, CNKI, and WANFANG databases. It was not restricted to year of publication up to April 2016 and language was restricted to Chinese or English.

### 2.3 Inclusion criteria

Studies were included if they met the following criteria: randomized or nonrandomized controlled study, age ≥18 years, posterior spinal surgery, articles on VTE after spine surgery, and patients with spine degeneration diseases.

### 2.4 Exclusion criteria

Studies were excluded if they met the following criteria: the factors that we could not extract from article for abnormal distribution or no specific number; patients with spinal trauma, spinal deformities, or tumors; abstracts, letters, reviews, or case reports; repeated data; and studies not reporting outcomes of interest.

### 2.5 Selection of studies

Two reviewers independently reviewed all subjects, abstracts, and the full text of articles. Then the eligible trials were selected according to the inclusion criteria. When consensus could not be reached, a third reviewer was consulted to resolve the disagreement.

### 2.6 Data extraction and management

Two reviewers extracted data independently. The data were extracted including the following categories: study ID, study design, study location, number of patients with VTE, total patients, demographic messages (age, sex, body mass index [BMI], history of drinking, smoking, hypertension [HT], heart disease [HD], and diabetes), and clinical outcomes (wearing elastic stockings, anticoagulant therapy, walking disability, surgical site [cervical vs lumbar], fusion vs nonfusion [lumbar fusion vs lumbar discectomy], surgical duration, and blood loss).

### 2.7 Statistical analysis

We used RevMan 5.3 (Nordic Cochrane Center, Cochrane Collaboration, Copenhagen, Denmark) and STATA 12.0 (Stata Corporation, College Station, TX) to analyze data and applied odds ratio (OR) and standardized mean difference (SMD), as summary statistics, to analyze dichotomous variables and continuous variables, respectively. Both were reported with 95% confidence intervals (CIs), and we regarded *P* < 0.05 as the level of statistical significance. Heterogeneity was tested using I^2^. If I^2^ >50%, it implied heterogeneity, and random-effects model was used; if I^2^ <50%, we chose fixed-effects model.

### 2.8 Test for risk of publication bias

We used funnel plot to assess publication bias. If there is publication bias, the funnel plot should be asymmetric, and if there is no publication bias, the funnel plot should be symmetric. Egger and Begg tests were used to measure the funnel plot asymmetry using a significance level of *P* < 0.05.

### 2.9 Sensitivity analysis overall

Because of the low heterogeneity of every factor, sensitivity analysis was not employed.

## 3 Results

### 3.1 Search results

**Figure 1:**
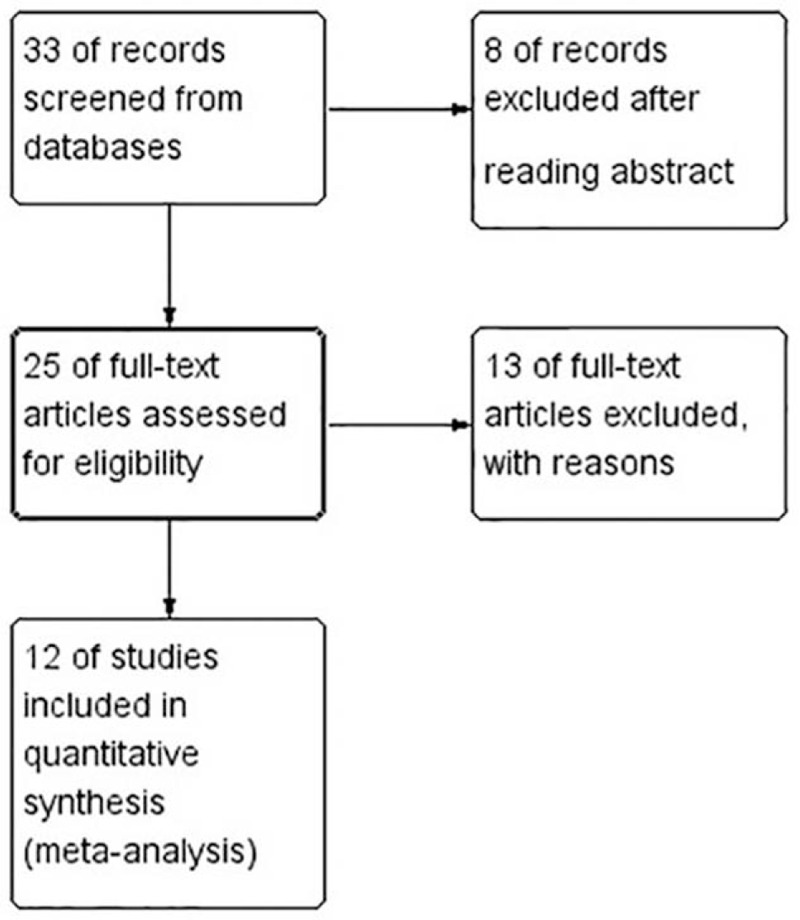
Flow diagram of study selection.

Twenty-seven English studies in MEDLINE and Embase and 6 Chinese studies in WANFANG and CNKI were searched. Of these, 6 English articles and 2 Chinese articles were excluded due to unrelated studies, either review articles or case reports, after our review of the abstract and titles. Another 12 English articles and 1 Chinese article were excluded due to data deficiency after our intensive reading of the full text. As a result, a total of 12 studies were identified for this meta-analysis. The literature search procedure is shown in Fig. 1.

### 3.2 Baseline characteristics and quality assessment

A total of 12 studies with 34,597 patients were identified, including 624 patients with VTE after spine surgery, and the incidence of VTE was 2% (0.4–14.4%). Baseline characteristics of the 2 groups are shown in Table 1.

**Table 1:**
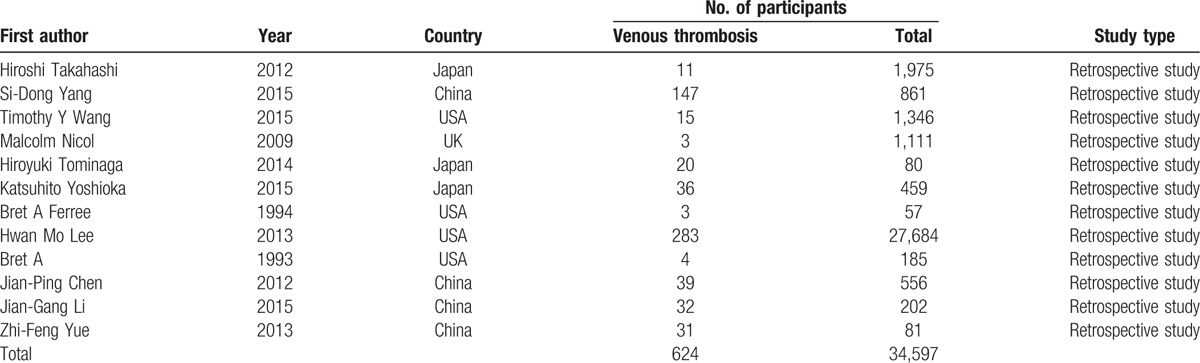
Characteristics of included studies.

As all included studies were retrospective studies, Newcastle Ottawa Quality Assessment Scale, maximum of 9 points, was used to assess the quality of each study. Nonrandomized case-controlled studies and cohort studies were performed by Newcastle Ottawa Quality Assessment Scale in term of selection, comparability, and exposure for study participants. The quality for each of our included studies was relatively high, because 8 scored 8 points and 4 scored 7 points (Table 2).

**Table 2:**
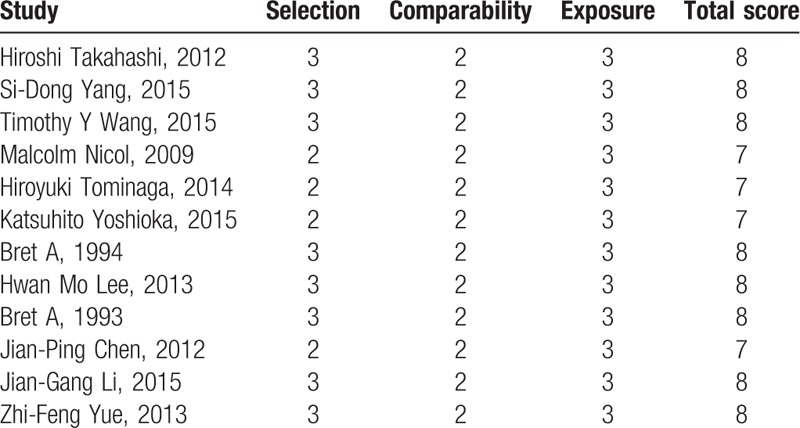
The quality assessment according to the Newcastle Ottawa Quality Assessment Scale (NOQAS) of each study.

### 3.3 Characteristics of patients

**Figure 2:**
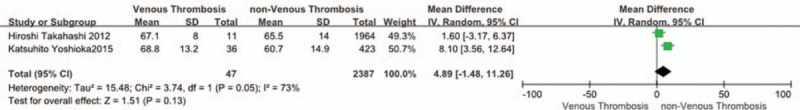
The standardized mean difference (SMD) estimate relationship between age and incidence of vein thrombosis after spine surgery. CI = confidence interval, df = degrees of freedom, SD = standard deviation.

Two studies^[4,28]^ reported relation between age at surgical time and incidence of VTE. The result showed that age at surgical time did not affect incidence of VTE (*P* = 0.13, SMD = 4.89 [−1.48, 11.26]; heterogeneity: *P* = 0.05, I^2^ = 73%, random-effects model; Fig. 2).

**Figure 3:**
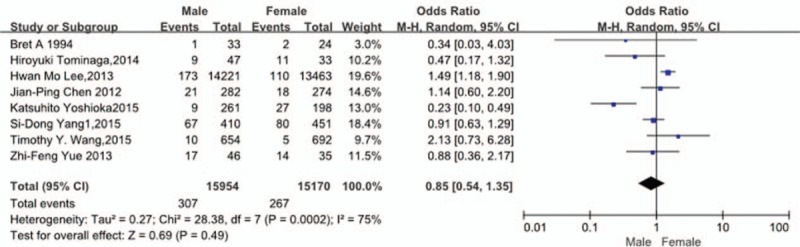
Forest plot showing the relationship between sex and the incidence of vein thrombosis after spine surgery. CI = confidence interval, df = degrees of freedom, M-H = Mantel–Haenszel.

Eight studies^[9,28–34]^ reported relation between sex and incidence of VTE. The result showed that sex did not affect incidence of VTE (*P* = 0.49, OR = 0.85, 95% CI [0.54, 1.35]; heterogeneity: *P* = 0.0002, I^2^ = 75%, random-effects model; Fig. 3).

**Figure 4:**
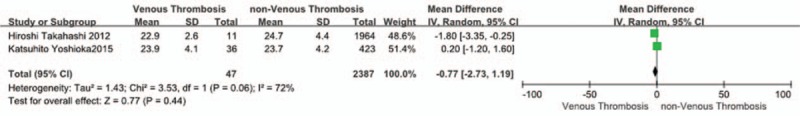
The standardized mean difference (SMD) estimate relationship between body mass index (BMI) and incidence of vein thrombosis after spine surgery. CI = confidence interval, df = degrees of freedom, SD = standard deviation.

Two studies^[4,28]^ reported that BMI of patients at the surgical time was negatively related to incidence of VTE. The result showed that BMI did not affect incidence of VTE (*P* = 0.44, SMD = −0.77 [−2.73, 1.19]; heterogeneity: *P* = 0.06, I^2^ = 72%, random-effects model; Fig. 4).

**Figure 5:**
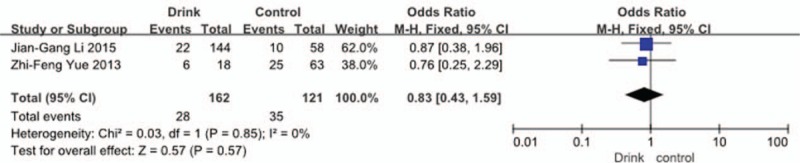
Forest plot showing the relationship between drinking and the incidence of vein thrombosis after spine surgery. CI = confidence interval, df = degrees of freedom, M-H = Mantel–Haenszel.

Two studies^[33,35]^ reported relation between history of drinking and incidence of VTE. The result showed that a history of drinking did not affect incidence of VTE (*P* = 0.57, OR = 0.83, 95% CI [0.43, 1.59]; heterogeneity: *P* = 0.85, I^2^ = 0%, fixed-effects model; Fig. 5).

**Figure 6:**
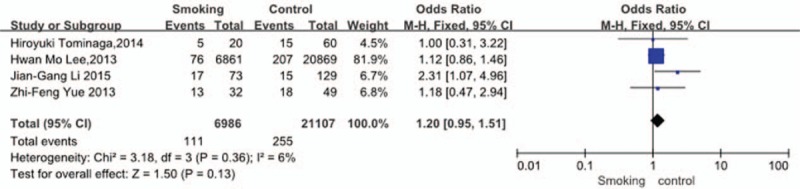
Forest plot showing the relationship between smoking and the incidence of vein thrombosis after spine surgery. CI = confidence interval, df = degrees of freedom, M-H = Mantel–Haenszel.

Four studies^[31,33–35]^ reported relation between history of smoking and incidence of VTE. The result showed that a history of smoking did not affect incidence of VTE (*P* = 0.13, OR = 1.20, 95% CI [0.95, 1.51]; heterogeneity: *P* = 0.36, I^2^ = 6%, fixed-effects model; Fig. 6).

**Figure 7:**
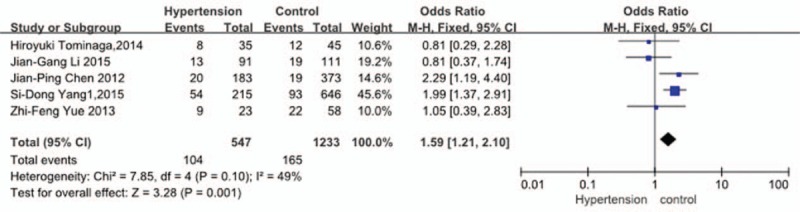
Forest plot showing the relationship between hypertension and the incidence of vein thrombosis after spine surgery. CI = confidence interval, df = degrees of freedom, M-H = Mantel–Haenszel.

Five studies^[30–33,35]^ reported relation between history of HT and incidence of VTE. The result showed that a history of HT significantly increased the incidence of VTE (*P* = 0.001, OR = 1.59, 95% CI [1.21, 2.10]; heterogeneity: *P* = 0.10, I^2^ = 49%, fixed-effects model; Fig. 7).

**Figure 8:**
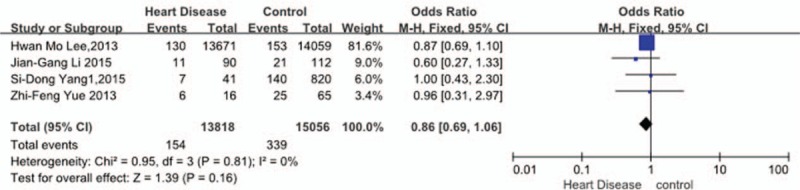
Forest plot showing the relationship between heart disease and the incidence of vein thrombosis after spine surgery. CI = confidence interval, df = degrees of freedom, M-H = Mantel–Haenszel.

Four studies^[30,33–35]^ reported relation between history of HD and incidence of VTE. The result showed that a history of HD did not affect incidence of VTE (*P* = 0.16, OR = 0.86, 95% CI [0.69, 1.06]; heterogeneity: *P* = 0.81, I^2^ = 0%, fixed-effects model; Fig. 8).

**Figure 9:**
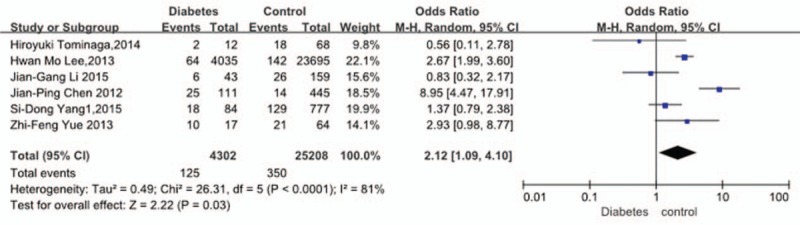
Forest plot showing the relationship between diabetes and the incidence of vein thrombosis after spine surgery. CI = confidence interval, df = degrees of freedom, M-H = Mantel–Haenszel.

Six studies^[30–35]^ reported relation between history of diabetes and incidence of VTE. The result showed that a history of diabetes significantly increased the incidence of VTE (*P* = 0.03, OR = 2.12, 95% CI [1.09, 4.10]; heterogeneity: *P* < 0.0001, I^2^ = 81%, random-effects model; Fig. 9).

### 3.4 Clinical outcomes

**Figure 10:**
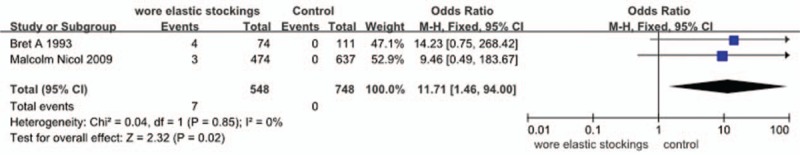
Forest plot showing the relationship between wearing elastic stockings and the incidence of vein thrombosis after spine surgery. CI = confidence interval, df = degrees of freedom, M-H = Mantel–Haenszel.

Two studies reported relation between wearing elastic stocking and incidence of VTE. The result showed that wearing elastic stocking significantly increased the incidence of VTE (*P* = 0.02, OR = 11.71, 95% CI [1.46, 94.00]; heterogeneity: *P* = 0.85, I^2^ = 0%, fixed-effects model; Fig. 10).

**Figure 11:**
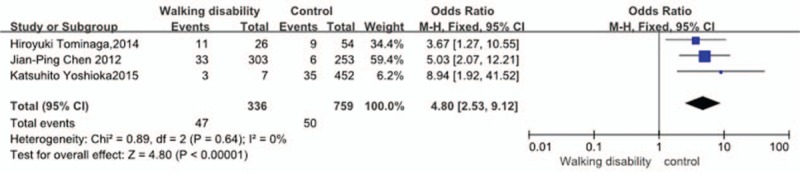
Forest plot showing the relationship between walking disability and the incidence of vein thrombosis after spine surgery. CI = confidence interval, df = degrees of freedom, M-H = Mantel–Haenszel.

Three studies^[28,31,32]^ reported relation between walking disability preoperation and incidence of VTE. The result showed that walking disability preoperation significantly increased the incidence of VTE (*P* < 0.00001, OR = 4.80, 95% CI [2.53, 9.12]; heterogeneity: *P* = 0.64, I^2^ = 0%, fixed-effects model; Fig. 11).

**Figure 12:**
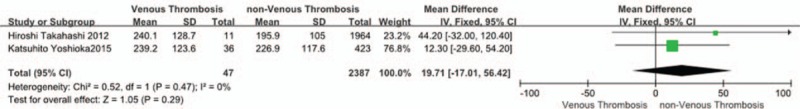
The standardized mean difference (SMD) estimate relationship between surgical duration and incidence of vein thrombosis after spine surgery. CI = confidence interval, df = degrees of freedom, SD = standard deviation.

Two studies^[4,28]^ reported relation between surgical duration and incidence of VTE. The result showed that surgical duration did not affect incidence of VTE (*P* = 0.29, SMD = 19.71 [−17.01, 56.42]; heterogeneity: *P* = 0.47, I^2^ = 0%, fixed-effects model; Fig. 12).

**Figure 13:**
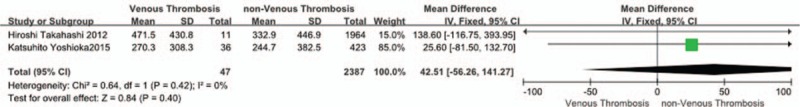
The standardized mean difference (SMD) estimate relationship between blood loss and incidence of vein thrombosis after spine surgery. CI = confidence interval, df = degrees of freedom, SD = standard deviation.

Two studies^[4,28]^ reported relation between blood loss and incidence of VTE. The result showed that blood loss did not affect incidence of VTE (*P* = 0.40, SMD = 42.51 [−56.26, 141.27]; heterogeneity: *P* = 0.42, I^2^ = 0%, fixed-effects model; Fig. 13).

**Figure 14:**
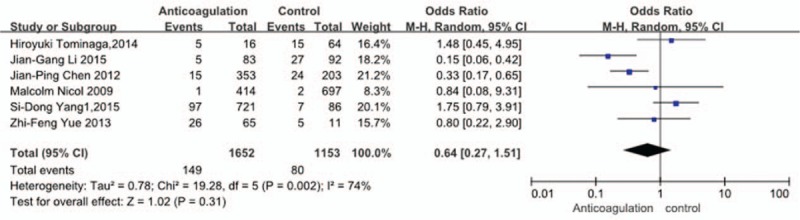
Forest plot showing the relationship between anticoagulant therapy and the incidence of vein thrombosis after spine surgery. CI = confidence interval, df = degrees of freedom, M-H = Mantel–Haenszel.

Six studies^[30–33,35,36]^ reported relation between anticoagulation therapy and incidence of VTE. The result showed that anticoagulation therapy did not affect incidence of VTE (*P* = 0.31, OR = 0.64, 95% CI [0.27, 1.51]; heterogeneity: *P* = 0.002, I^2^ = 74%, random-effects model; Fig. 14).

**Figure 15:**
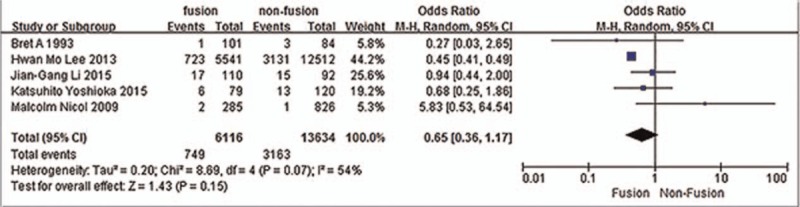
Forest plot showing the relationship between lumbar fusion and the incidence of vein thrombosis after spine surgery. CI = confidence interval, df = degrees of freedom, M-H = Mantel–Haenszel.

Five studies^[28,34–37]^ reported relation between lumbar fusion and incidence of VTE. The result showed that lumbar fusion did not affect incidence of VTE (*P* = 0.15, OR = 0.65, 95% CI [0.36, 1.17]; heterogeneity: *P* = 0.07, I^2^ = 54%, random-effects model; Fig. 15).

**Figure 16:**
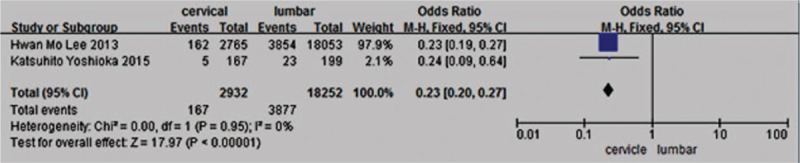
Forest plot showing the relationship between lumbar surgery and the incidence of vein thrombosis after spine surgery. CI = confidence interval, df = degrees of freedom, M-H = Mantel–Haenszel.

Two studies^[28,34]^ reported relation between surgery site (cervical vs lumbar) and incidence of VTE. The result showed that lumbar surgery significantly increased the incidence of VTE (*P* < 0.00001, OR = 0.23, 95% CI [0.20, 0.27]; heterogeneity: *P* = 0.95, I^2^ = 0%, fixed-effects model; Fig. 16).

### 3.5 Publication bias

After a detection of publication bias by STATA 12.0, no publication bias was found for all included studies (all *P* > 0.05). The funnel plot did not indicate any publication bias in sex (Begg, *P* = 0.621; Egger, *P* = 0.173), drinking (Begg, *P* = 0.317), smoking (Begg, *P* = 1.000; Egger, *P* = 0.631), HT (Begg, *P* = 0.142; Egger, *P* = 0.177), HD (Begg, *P* = 0.497; Egger, *P* = 0.910), diabetes (Begg, *P* = 0.573; Egger, *P* = 0.448), wearing elastic stockings (Begg, *P* = 0.317), anticoagulant therapy (Begg, *P* = 0.851; Egger, *P* = 0.711), walking disability postoperation (Begg, *P* = 0.602; Egger, *P* = 0.806), and fusion (Begg, *P* = 0.327; Egger, *P* = 0.171).

## 4 Discussion

As is known to all, in the orthopedics field, the rate for VTE varies according to the pathology, surgical site, or surgical options. Compared with upper limb surgery,^[38]^ lower limb surgery such as hip or knee arthroplasty has a high risk of VTE.^[8–10]^ Recent studies focused on VTE after spinal operation; however, the incidence and risk factors of VTE postoperation remain undebated. A previous meta-analysis focused on the prevalence of VTE after spinal surgery,^[29]^ but there is none that focuses on the risk factors for this prevalence. As we know, this is the first meta-analysis to summarize and analyze the risk factors of VTE after spine surgery. And we also evaluate efficacy of low-molecular-weight heparin after spine surgery and observe difference in incidence of VTE between Asians and the Occidentals. The results showed that Asian ethnicity, walking disability preoperation, wearing elastic stocking, lumbar surgery, history of HT, and diabetes were risk factors for VTE after spine surgery.

A total of 34,597 patients from 12 articles were included in our study, including 624 patients with VTE after spine surgery; the incidence of VTE was 2%. Moreover, we surprisingly found that in 316 of 4214 Asian patients with VTE, the incidence of VTE was 7.5%; however, compared with 308 of 30,383 Occidental patients with VTE, the incidence of VTE was 1%. The difference was significant (*P* < 0.0001), which implied that the Asian patients were more likely to suffer from VTE after spine surgery, but the reason was still unclear.

Nicol et al^[36]^ and Ferree and Wright^[37]^ explored whether wearing elastic stocking increased the incidence of VTE after spine surgery or not. Our result implied that patients who wore elastic stocking after operation were more likely to suffer from VTE, compared with those who did not wear them (*P* = 0.02). Our result was opposite to prevention guideline for VTE, but the reason was still unclear. We need prospective randomized controlled trials (RCTs), multicenter studies, and a big sample to demonstrate whether wearing elastic stocking increases the incidence of VTE after spine surgery or not.

Five studies^[30–33,35]^ reported the relation between history of HT and incidence of VTE. Yang et al^[30]^ proved that a history of HT significantly increased the incidence for VTE, but Tominaga et al^[31]^ had an idea that was opposite of that of Yang et al. In our analysis, the result showed that a history of HT significantly increased the incidence for VTE (*P* = 0.001). We had a hypothesis that the vascular elasticity was usually not well for the patients with HT, which may affect hemodynamics and blood coagulation mechanism, and this may be related to higher incidence of VTE postoperation.

Six studies^[30–35]^ reported relation between history of diabetes and incidence of VTE. Yang et al^[30]^ and Tominaga et al^[31]^ believed that a history of diabetes was not an important key for VTE, but Herzog and coworkers^[34]^ had an opposite idea. In our analysis, the result showed that a history of diabetes significantly increased the incidence for VTE (*P* = 0.03). The fact that diabetes, as an independent factor, significantly increased the incidence of VTE has never been highlighted in previous studies. But our results indicated that we must control blood glucose of patients preoperation and postoperation, especially postoperation. Higher blood glucose and endocrine metabolic disturbances for patients may cause hormone imbalance for the whole body, which may lead to patients with diabetes to suffer from VTE easily.

Three studies^[3,19,23]^ reported relation between walking disability preoperation and incidence of VTE. In our analysis, the result showed that walking disability preoperation significantly increased the incidence for VTE (*P* < 0.00001). Having walking disability preoperation means that lower limbs have a poor disability of muscle contraction function, leading to slow blood flow in lower extremities, due to which VTE occurs easily. In clinic, attention must be paid to patients with walking disability postoperation and measures must be taken to prevent incidence of VTE.

Two studies^[28,34]^ reported relation between surgery site (cervical vs lumbar) and incidence of VTE. The result showed that lumbar surgery significantly increased the incidence of VTE (*P* < 0.00001). Patients who underwent cervical surgery left bed earlier postoperation, compared with patients who underwent lumbar surgery. The results showed that leaving the bed to do postoperative functional exercise early was beneficial to prevent incidence of VTE.

Six studies^[30–33,35,36]^ explored the relation between anticoagulation therapy and VTE. Some authors believed that anticoagulant therapy could lower incidence of DVT. Shepherd and Mills^[14]^ reported that the use of pharmacological prophylaxis significantly reduced the prevalence of DVT compared with mechanical prophylaxis or no prophylaxis. But Yang et al^[30]^ and Tominaga et al^[31]^ disagreed with it. Our result showed that anticoagulation therapy could not decrease the risk of VTE (*P* = 0.31). Yang et al^[30]^ reported that it may be related to the dosage of anticoagulation. The reason why anticoagulation therapy could not decrease risk of VTE has not been clear.

Some studies reported that other factors including age, sex, BMI, history of drinking, smoking, HD, surgical duration, lumbar fusion, and blood loss were risk factors of VTE after spine surgery, but some were not. In our analysis, these were not risk factors of VTE after spine surgery. Regarding surgical duration and blood loss, we may consider that they can cause blood to be in a state of high condensation, which can easily lead to the formation of blood clots. But in our study, both were not risk factors for VTE. Not enough surgical duration and blood loss may cause these results. Further studies are needed to determine whether longer surgical duration and more blood loss are risk factors of VTE. Even if surgical duration and blood loss remain the same, old people, compared with young people, tended to suffer more from VTE. But the data we collected were not significantly different.

### 4.1 Study limitations

This study has several limitations. First, we could not perform subgroup analysis or single spine degeneration disease analysis due to few included articles. Some factors that we considered initially, such as D-dimer and surgical time >2 versus <2 hours, could not be extracted. Second, we mainly explored the prevalence and risk factors of VTE in lumbar and cervical degeneration diseases regardless of other spine diseases. Finally, none of the studies included in the meta-analysis was a RCT. Although our study has limitations, to our knowledge, this is the first meta-analysis to explore risk factors of VTE after spine surgery.

In summary, we found that Asian patients, patients with walking disability preoperation, patients wearing elastic stocking, patients with a history of HT, patients having undergone lumbar surgery, and patients experiencing diabetes significantly increased the incidence of VTE after spine surgery. To provide objective data on the clinical results of both procedures, a well-designed, prospective RCT should be performed in the future.

## References

[1] Bang SM Jang MJ Oh D Korean guidelines for the prevention of venous thromboembolism. J Korean Med Sci 2010;25:1553–9. 21060742 10.3346/jkms.2010.25.11.1553 PMC2966990

[2] Koo KH Choi JS Ahn J Comparison of clinical and physiological efficacies of different intermittent sequential pneumatic compression devices in preventing deep vein thrombosis: a prospective randomized study. Clin Orthop Surg 2014;6:468–75. 25436073 10.4055/cios.2014.6.4.468 PMC4233228

[3] Strom RG Frempong-Boadu AK Low-molecular-weight heparin prophylaxis 24 to 36 hours after degenerative spine surgery: risk of hemorrhage and venous thromboembolism. Spine (Phila Pa 1976) 2013;38:E1498–502. 23873245 10.1097/BRS.0b013e3182a4408d

[4] Takahashi H Yokoyama Y Iida Y Incidence of venous thromboembolism after spine surgery. J Orthop Sci 2012;17:114–7. 22222443 10.1007/s00776-011-0188-2

[5] Anderson FA Jr Spencer FA Risk factors for venous thromboembolism. Circulation 2003;107 (23 suppl 1):16–9. 10.1161/01.CIR.0000078469.07362.E6 12814980

[6] Yoshioka K Kitajima I Kabata T Venous thromboembolism after spine surgery: changes of the fibrin monomer complex and D-dimer level during the perioperative period. J Neurosurg Spine 2010;13:594–9. 21039150 10.3171/2010.5.SPINE09883

[7] Freedman KB Brookenthal KR Fitzgerald RH Jr A meta analysis of thromboembolic prophylaxis following elective total hip arthroplasty. J Bone Joint Surg Am 2000;82:929–38. 10901307 10.2106/00004623-200007000-00004

[8] Righini M Bounameaux H Clinical relevance of distal deep vein thrombosis. Curr Opin Pulm Med 2008;14:408–13. 18664970 10.1097/MCP.0b013e32830460ea

[9] Wang TY Sakamoto JT Nayar G Independent predictors of 30-day perioperative deep vein thrombosis in 1346 consecutive patients after spine surgery. World Neurosurg 2015;84:1605–12. 26171892 10.1016/j.wneu.2015.07.008

[10] Xing KH Morrison G Lim W Has the incidence of deep vein thrombosis in patients undergoing total hip/knee arthroplasty changed over time? A systematic review of randomized controlled trials. Thromb Res 2008;123:24–34. 18620740 10.1016/j.thromres.2008.05.005

[11] Lieberman JR Geerts WH Current concept review: prevention of venous thromboembolism after total hip and knee arthroplasty. J Bone Joint Surg Am 1994;76:1239–50. 8056805 10.2106/00004623-199408000-00015

[12] Maung AA Schuster KM Kaplan LJ Risk of venous thromboembolism after spinal cord injury: not all levels are the same. J Trauma 2011;71:1241–5. 22071925 10.1097/TA.0b013e318235ded0

[13] Hansson PO Sörbo J Eriksson H Recurrent venous thromboembolism after deep vein thrombosis: incidence and risk factors. Arch Intern Med 2000;160:769–74. 10737276 10.1001/archinte.160.6.769

[14] Shepherd A Mills C Fatal pulmonary embolism following hip and knee replacement. A study of 2153 cases using routine mechanical prophylaxis and selective chemoprophylaxis. Hip Int 2006;16:53–6. 19219778 10.5301/hip.2008.4713

[15] Rokito SE Schwartz MC Neuwirth MG Deep vein thrombosis after major reconstructive spinal surgery. Spine 1996;21:853–9. 8779018 10.1097/00007632-199604010-00016

[16] Oda T Fuji T Kato Y Deep venous thrombosis after posterior spinal surgery. Spine 2000;25:2962–7. 11074685 10.1097/00007632-200011150-00019

[17] Leon L Rodriguez H Tawk RG The prophylactic use of inferior vena cava filters in patients undergoing high-risk spinal surgery. Ann Vasc Surg 2005;19:442–7. 15864473 10.1007/s10016-005-0025-1

[18] Amin BY Tu TH Schairer WW Pitfalls of calculating hospital readmission rates based on nonvalidated administrative data sets. J Neurosurg Spine 2013;18:134–8. 23186376 10.3171/2012.10.SPINE12559

[19] Epstein NE Intermittent pneumatic compression stocking prophylaxis against deep venous thrombosis in anterior cervical spinal surgery: a prospective efficacy study in 200 patients and literature review. Spine 2005;30:2538–43. 16284592 10.1097/01.brs.0000186318.80139.40

[20] Buerba RA Giles E Webb ML Increased risk of complications after anterior cervical discectomy and fusion in the elderly: an analysis of 6253 patients in the American College of Surgeons National Surgical Quality Improvement Program database. Spine (Phila Pa 1976) 2014;39:2062–9. 25271519 10.1097/BRS.0000000000000606

[21] Yoshioka K Murakami H Demura S The prevalence of and specific risk factors for venous thromboembolic disease following elective spine surgery. J Bone Joint Surg Am 2010;92:304–13. 20124056 10.2106/JBJS.H.01815

[22] Goldhaber SZ Risk factors for venous thromboembolism. J Am Coll Cardiol 2010;56:1–7. 20620709 10.1016/j.jacc.2010.01.057

[23] Falck-Ytter Y Francis CW Johanson NA Prevention of VTE in orthopedic surgery patients: Antithrombotic Therapy and Prevention of Thrombosis, 9th ed: American College of Chest Physicians Evidence-Based Clinical Practice Guidelines. Chest 2012;141 (2 suppl):e278S–325S. 22315265 10.1378/chest.11-2404 PMC3278063

[24] Pugely AJ Martin CT Gao Y Outpatient surgery reduces short-term complications in lumbar discectomy: an analysis of 4310 patients from the ACS-NSQIP database. Spine 2013;38:264–71. 22814304 10.1097/BRS.0b013e3182697b57

[25] Paiement GD Wessinger SJ Harris WH Cost-effectiveness of prophylaxis in total hip replacement. Am J Surg 1991;161:519–24. 1903606 10.1016/0002-9610(91)91124-2

[26] Kearon C Natural history of venous thromboembolism. Circulation 2003;107:I22–30. 12814982 10.1161/01.CIR.0000078464.82671.78

[27] Lee HM Suk KS Moon SH Deep vein thrombosis after major spinal surgery: incidence in an East Asian population. Spine (Phila Pa 1976) 2000;25:1827–30. 10888952 10.1097/00007632-200007150-00014

[28] Yoshioka K Murakami H Demura S Prevalence and risk factors for development of venous thromboembolism after degenerative spinal surgery. Spine (Phila Pa 1976) 2015;40:E301–6. 25494320 10.1097/BRS.0000000000000727

[29] Ferree BA Deep venous thrombosis following lumbar laminotomy and laminectomy. Orthopedics 1994;17:35–8. 8121832 10.3928/0147-7447-19940101-06

[30] Yang SD Liu H Sun YP Prevalence and risk factors of deep vein thrombosis in patients after spine surgery: a retrospective case-cohort study. Sci Rep 2015;5:11834. 26135271 10.1038/srep11834 PMC4488742

[31] Tominaga H Setoguchi T Tanabe F Risk factors for venous thromboembolism after spine surgery. Medicine (Baltimore) 2015;94:e466. 25654385 10.1097/MD.0000000000000466 PMC4602703

[32] Chen J-P Qian R Zhang T Analysis on risk factors of venous thromboembolism after spine surgery. Mod Prev Med 2012;39:5705–8.

[33] Yue Z-F Chen J-Y Chai W Analysis on affecting factors of lower extremity deep venous thromboembolism after spine operation. Chin J Clinicians (Electronic Edition) 2013;7:5840–3.

[34] Schoenfeld AJ Herzog JP Dunn JC Patient-based and surgical characteristics associated with the acute development of deep venous thrombosis and pulmonary embolism after spine surgery. Spine (Phila Pa 1976) 2013;38:1892–8. 23778367 10.1097/BRS.0b013e31829fc3a0

[35] Li G-J Zhao X Logistic regression analysis on risk factors of deep venous thromboembolism in spinal operation. Hainan Med J 2015;26:658–60.

[36] Nicol M Sun Y Craig N Incidence of thromboembolic complications in lumbar spinal surgery in 1,111 patients. Eur Spine J 2009;18:1548–52. 19484271 10.1007/s00586-009-1035-4 PMC2899370

[37] Ferree BA Wright AM Deep venous thrombosis following posterior lumbar spinal surgery. Spine (Phila Pa 1976) 1993;18:1079–82. 8367776 10.1097/00007632-199306150-00019

[38] Jameson SS James P Howcroft DW Venous thromboembolic events are rare after shoulder surgery: analysis of a national database. J Shoulder Elbow Surg 2011;20:764–70. 21420324 10.1016/j.jse.2010.11.034

